# Supplementary material for: Semaglutide combined with empagliflozin vs. monotherapy for non-alcoholic fatty liver disease in type 2 diabetes: Study protocol for a randomized clinical trial
Source: PLoS One. 2024 May 3;19(5):e0302155. doi: 10.1371/journal.pone.0302155 (PMC11068176; doi:10.1371/journal.pone.0302155)
Supplement: S1 File — (DOCX) [file pone.0302155.s002.docx]

**临床试验研究计划书**

**Research Protocol**

**项目名称：司美格鲁肽和恩格列净联合与单药治疗2型糖尿病伴非酒精性脂肪性肝病疗效和安全性比较**

**负 责 人：林宇豪**

**承担单位：厦门弘爱医院**

**目录**

[一、诚信申明 3](#_Toc153036321)

[二、经费来源： 3](#_Toc153036322)

[三、研究背景 3](#_Toc153036323)

[四、研究目的 5](#_Toc153036324)

[五、纳入和排除标准 5](#_Toc153036325)

[六、设计方案 5](#_Toc153036326)

[七、样本量估算： 7](#_Toc153036327)

[八、随机和隐蔽分组的方法 7](#_Toc153036328)

[九、盲法 7](#_Toc153036329)

[十、测量指标 7](#_Toc153036330)

[十一、对参试者有效性认定的定义 8](#_Toc153036331)

[十二、不良事件和不良反应的定义、鉴定方法和管理制度 8](#_Toc153036332)

[十三、参试者的征募 8](#_Toc153036333)

[十四、参试者一般信息的收集 8](#_Toc153036334)

[十五、统计分析方法 8](#_Toc153036335)

[十六、管理制度 9](#_Toc153036336)

[十七、试验结束后对参试者的治疗和管理 9](#_Toc153036337)

# 一、诚信申明

本课题组郑重声明，本研究操作过程均严格按照课题方案进行，真实准确记录试验数据。本课题组所产生的相关研究成果，包括技术标准、专利等相关知识产权归属课题组所有。课题组完全意识到本声明应承担的法律责任。本课题组不存在利益冲突。

# 二、经费来源：

厦门市医疗卫生指导性项目（3502Z20224ZD1106）

# 三、研究背景

糖尿病是一组由于胰岛素分泌和（或）利用缺陷引起，以慢性高血糖为特征的代谢性疾病。非酒精性脂肪肝是与肥胖及代谢功能障碍相关的肝脏疾病，其与糖尿病密切相关。对东亚人群的流行病学调查显示，糖尿病人群中的非酒精性脂肪肝患病率为55%，严重危害人类健康并对社会造成巨大经济负担^(1)^。

2型糖尿病常与非酒精性脂肪肝共存，且2型糖尿病是加速非酒精性脂肪肝进展的重要因素。2型糖尿病会降低肝脏组织对于胰岛素作用的敏感性，从而导致胰岛素抵抗；而胰岛素抵抗被认为是非酒精性脂肪肝发病的中心环节^(2)^。胰岛素抵抗会驱动肝脏脂肪从头合成，导致游离脂肪酸水平的增加，从而提高机体对游离脂肪酸的利用，以致肝脏、胰腺产生多余的脂肪细胞并形成脂肪肝及脂肪胰，而脂肪肝及脂肪胰可通过多种途径导致β细胞功能缺陷和胰岛素抵抗，以此形成负性循环^(3)^。因此，推测通过稳定2型糖尿病伴非酒精性脂肪性肝病患者的糖脂代谢水平,可以达到控制疾病进展的效果。

现阶段，对于非酒精性脂肪性肝病的治疗尚无特效药，而胰高血糖素样肽-1受体激动剂和钠-葡萄糖协同转运蛋白2抑制剂作为降糖药，同时具有减重及改善肝脏脂肪变性的作用^(4)^。而且，真实世界数据分析发现，胰高血糖素样肽-1受体激动剂和钠-葡萄糖协同转运蛋白2抑制剂能显著改善2型糖尿病伴非酒精性脂肪肝患者的肝脏脂肪变性和纤维化^(5)^。一项对于2型糖尿病伴非酒精性脂肪肝的研究发现，与对照组相比，胰高血糖素样肽-1受体激动剂度拉糖肽可以显著降低肝内脂肪含量，其作用与体重、血糖的下降并无显著相关性^(6)^。另一项对于非酒精性肝炎的研究发现，与对照组相比，胰高血糖素样肽-1受体激动剂司美格鲁肽可以改善肝脏脂肪变性以及血糖水平^(7)^。还有研究发现，钠-葡萄糖协同转运蛋白2抑制剂恩格列净可以显著降低2型糖尿病伴非酒精性脂肪肝患者的肝内脂肪含量以及血糖水平，该研究还发现恩格列净对于肝内脂肪含量的作用与血糖及体重的下降无显著相关性^(8)^。

目前，对于胰高血糖素样肽-1受体激动剂联合钠-葡萄糖协同转运蛋白2抑制剂联合治疗非酒精性脂肪肝患者的研究甚少。一项对于2型糖尿病的研究发现，胰高血糖素样肽-1受体激动剂和钠-葡萄糖协同转运蛋白2抑制剂联合使用，可以降低肝脏相关生物指标以及改善肝脏脂肪变性^(9)^。我们推测，司美格鲁肽与恩格列净联合使用可以改善2型糖尿病伴非酒精性脂肪性肝病患者的肝脏脂肪变性以及血糖的控制。

虽然目前有研究表明，胰高血糖素样肽-1受体激动剂与钠-葡萄糖协同转运蛋白2抑制剂可以降低非酒精性脂肪肝的肝内脂肪含量，延缓疾病进展。但是，其作用机制仍未完全阐明。有研究发现，胰高血糖素可以诱导氨基酸分解代谢，从而促进肝脏β氧化以及胰岛素分泌，减少脂肪生成，并降低循环游离脂肪酸浓度，减少脂肪在肝脏及胰腺的蓄积^(10, 11)^。而非酒精性脂肪肝则会损害胰高血糖素诱导的氨基酸分解代谢，因此，我们推测司美格鲁肽、恩格列净可以通过调节胰高血糖素对氨基酸及游离脂肪酸的作用，从而延缓非酒精性脂肪性肝病的进展。

**主要参考文献：**

1. Z. M. Younossi et al., The global epidemiology of NAFLD and NASH in patients with type 2 diabetes: A systematic review and meta-analysis. J Hepatol 71, 793-801 (2019).

2. S. L. Friedman, B. A. Neuschwander-Tetri, M. Rinella, A. J. Sanyal, Mechanisms of NAFLD development and therapeutic strategies. Nat Med 24, 908-922 (2018).

3. F. Gerst et al., Metabolic crosstalk between fatty pancreas and fatty liver: effects on local inflammation and insulin secretion. Diabetologia 60, 2240-2251 (2017).

4. A. Mantovani, C. D. Byrne, G. Targher, Efficacy of peroxisome proliferator-activated receptor agonists, glucagon-like peptide-1 receptor agonists, or sodium-glucose cotransporter-2 inhibitors for treatment of non-alcoholic fatty liver disease: a systematic review. Lancet Gastroenterol Hepatol 7, 367-378 (2022).

5. S. Colosimo et al., Effects of antidiabetic agents on steatosis and fibrosis biomarkers in type 2 diabetes: A real-world data analysis. Liver Int 41, 731-742 (2021).

6. M. S. Kuchay et al., Effect of dulaglutide on liver fat in patients with type 2 diabetes and NAFLD: randomised controlled trial (D-LIFT trial). Diabetologia 63, 2434-2445 (2020).

7. P. N. Newsome et al., A Placebo-Controlled Trial of Subcutaneous Semaglutide in Nonalcoholic Steatohepatitis. N Engl J Med 384, 1113-1124 (2021).

8. M. S. Kuchay et al., Effect of Empagliflozin on Liver Fat in Patients With Type 2 Diabetes and Nonalcoholic Fatty Liver Disease: A Randomized Controlled Trial (E-LIFT Trial). Diabetes Care 41, 1801-1808 (2018).

9. A. Gastaldelli et al., Exenatide and dapagliflozin combination improves markers of liver steatosis and fibrosis in patients with type 2 diabetes. Diabetes Obes Metab 22, 393-403 (2020).

10. K. M. Habegger, Cross Talk Between Insulin and Glucagon Receptor Signaling in the Hepatocyte. Diabetes 71, 1842-1851 (2022).

11. M. M. Richter et al., The Liver-α-Cell Axis in Health and in Disease. Diabetes 71, 1852-1861 (2022).

# 四、研究目的

本项目拟通过临床试验，将2型糖尿病伴非酒精性脂肪性肝病患者随机分为三组，分别予以司美格鲁肽、恩格列净、司美格鲁肽+恩格列净，研究各组对肝内脂肪含量、糖尿病缓解率以及体重的影响。并使用胃肠道不适症状发生率、生殖道感染发生率来评估三组之间的安全性差异。并进一步测定胰高血糖素及游离脂肪酸，探究司美格鲁肽和恩格列净在非酒精性脂肪性肝病中的作用机制，以期为临床应用提供理论依据。

# 五、纳入和排除标准

（一）纳入标准：

1.在入组是年龄≥18岁

2.脂肪肝

3.2型糖尿病

3.超重或肥胖（BMI≥24kg/m^2^）

4.入组前4周未使用胰高血糖素样肽-1受体激动剂和钠-葡萄糖协同转运蛋白2抑制剂。

（二）排除标准

1.1型糖尿病，妊娠期糖尿病或其他特殊类型糖尿病；

2.既往服用过噻唑烷二酮类降糖药；

3.合并心、肝、肾功能不全；

4.合并病毒性肝炎（比如：乙型病毒性肝炎），酒精性肝炎，自身免疫性肝炎，药物性肝炎，血色素沉着症，Wilson’s 疾病，肝硬化，先天性代谢性疾病（比如：胆固醇脂沉积病）或其他导致慢性肝病的原因；

5.合并脑卒中、恶性肿瘤、胰腺炎；

6.合并甲状腺髓样癌或2型多发性内分泌腺瘤个人史或家族史；

7.妊娠、哺乳及在试验期间预期妊娠；

8.男性每周饮酒量＞140g，女性每周饮酒量＞70g；

9.既往胰高血糖素样肽-1受体激动剂、钠-葡萄糖协同转运蛋白2抑制剂、二甲双胍过敏史；

10.精神疾病史；

11.慢性贫血病史（男性血红蛋白＜100g/L，女性血红蛋白＜90g/L）；

12．不愿意或不能签署知情同意书。

# 六、设计方案

以2型糖尿病伴非酒精性脂肪性肝病患者为研究对象，采用前瞻性随机对照研究设计，观察105例患者。根据纳入、排除标准，将纳入病例随机分为司美格鲁肽组（代码A）、恩格列净组（代码B）、司美格鲁肽+恩格列净组（代码C），干预时间为52周。在入组当天、干预后第12周、第24周、第52周进行相关指标的测定。具体技术路线图如下：


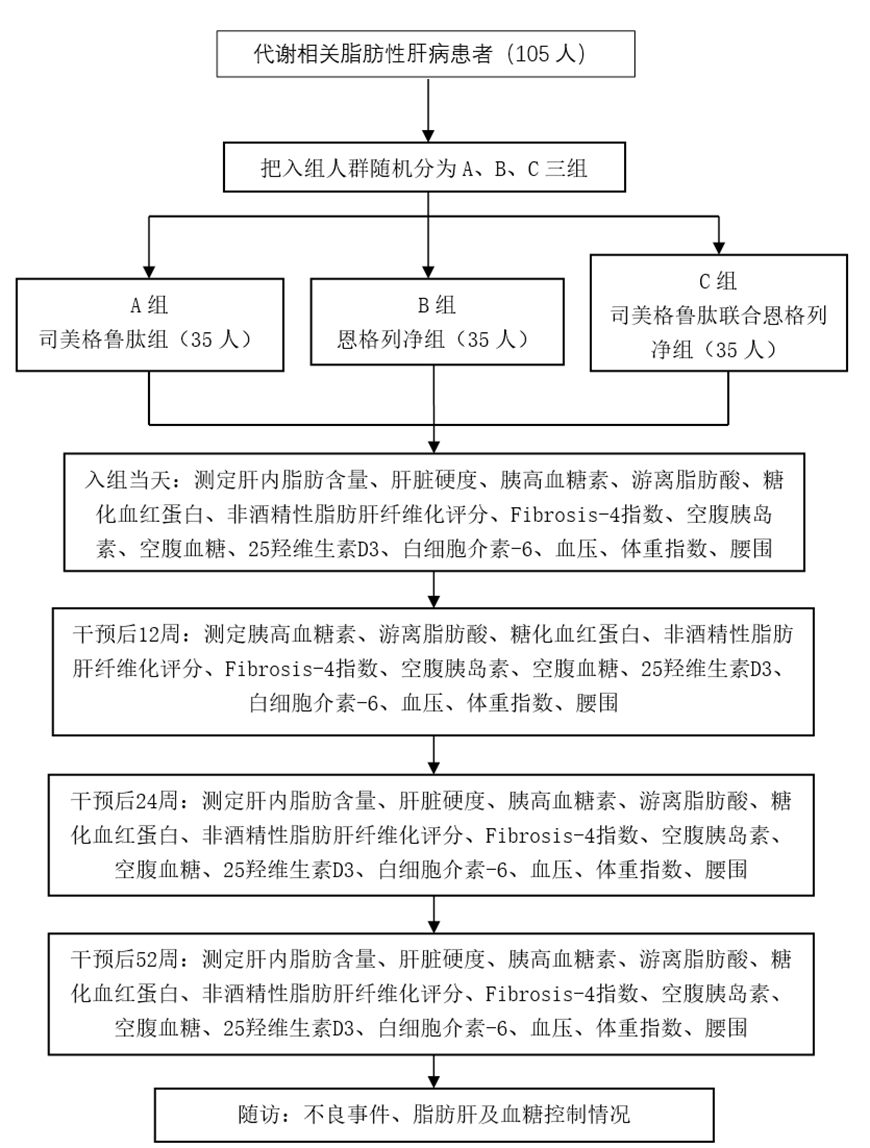

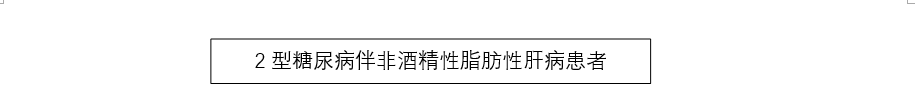


# 七、样本量估算：

取α=0.05，设定把握度为Power=0.90，采用NCSS-PASS中基于均值的单因素方差分析（One-way Analysis of Variance）计算样本量，每组需要(8+27+58)/3=31，考虑到脱落因素，脱落率按10%估计，每组需要31/(1-0.1)=35例，共需要35*3=105例。

# 八、随机和隐蔽分组的方法

由统计中心根据电脑随机数字分组。随机号以密闭不透光牛皮纸信封隐藏。

# 九、盲法

对研究人员及病人设置盲法。

# 十、测量指标

（一）主要指标：

1.脂肪肝程度：通过肝脏超声在第0、24、52周测定；

2.胰高血糖素：通过ELISA在第0、12、24、52周测定；

3.游离脂肪酸：通过ELISA在第0、12、24、52周测定；

（二）次要指标：

1.肝脏硬度：通过肝脏超声在第0、24、52周测定；

2.非酒精性脂肪肝纤维化评分：通过公式第0、12、24、52周计算所得；

3.Fibrosis-4指数：通过公式第0、12、24、52周计算所得；

4.空腹胰岛素：通过直接化学发光法在第0、12、24、52周测定；

5.空腹血糖：通过酶化学法在第0、12、24、52周测定；

6.糖化血红蛋白：通过高效液相层析法在第0、12、24、52周测定；

7.25羟维生素D3：通过酶化学法在第0、12、24、52周测定；

8.白细胞介素-6：通过ELISA法在第0、12、24、52周测定；

9.铁蛋白：通过化学发光免疫分析法在第0、12、24、52周测定；

10.血压：通过血压计在第0、12、24、52周测量；

11.BMI：通过公式在第0、12、24、52周计算所得；

12.脂联素：通过ELISA法在第0、12、24、52周测定；

（三）安全性指标：

实时观察患者胃肠道不适症状、生殖道感染症状，在12周、24周、52周进行统计发生不良事件的人数，并进行率计算。

# 十一、对参试者有效性认定的定义

1.受试者有权在临床试验的任何阶段随时退出试验。

2.当试验期间出现以下情况主动让受试者退出临床试验：

I.试验期间出现不可耐受胰高血糖素样肽-1受体激动剂或钠-葡萄糖协同转运蛋白2抑制剂；

II.试验期间出现糖尿病急性并发症；

III.试验期间未能按时服药；

IV.试验期间因各种原因不能按时完成检验检查；

3.研究者将自己的联系方式主动告知受试者，并主动获取受试者的最新联系方式。

# 十二、不良事件和不良反应的定义、鉴定方法和管理制度

1.不良事件为患者或临床研究受试者在接受研究干预时发生的不必一定与该治疗干预有因果联系的任何不利的医学事件。

2.当患者出现胰腺炎、恶心、呕吐等胃肠道反应、泌尿道感染则定义为不良反应。

3.当出现不良反应时根据患者耐受情况及不良反应的严重程度，予以对症治疗、停止用药等方式。

# 十三、参试者的征募

征募地点为厦门弘爱医院。

招聘公告在医院内部公告栏、微信朋友圈、微信群、微博等社交媒体平台上发布。潜在参与者的资格将由研究协调员评估。之后，研究协调员将根据纳入和排除标准筛选符合条件的参与者。如果所有参与者同意参加本研究，他们将被要求签署一份知情同意书。

# 十四、参试者一般信息的收集

厦门弘爱医院内分泌科医生收集参试者的手机号码、微信、家庭住址、主要联系人的联系方式。

# 十五、统计分析方法

1.符合正态分布的定量数据使用t检验来验证两组数据的差异性。

2.符合正态分布的定量数据使用方差分析来验证多组数据的差异性。

3.定性数据使用卡方检验来验证各组数据之间的差异性。

4.不符合正态分布的定量数据使用Kruskal-Wallis检验来验证各组数据之间的差异性。

5.Logistics回归分析来分析变量之间的相关性。

当试验过程中因各种原因出现人员减少时，通过ITT分析结局指标。

# 十六、管理制度

让参试者在入组时加入微信群，由专业人员进行统一管理。

对于试验标本由厦门弘爱医院内分泌科专业护士进行采集，由厦门弘爱医院检验科进行检验及保存。

数据采集由专业人员进行采集、记录，并保存在Excel表格中，由另外一人进行核对。

# 十七、试验结束后对参试者的治疗和管理

试验结束后若参试者脂肪肝及血糖仍未获改善，则进行方案的更改，包括但不限于：增加降糖药物、胰岛素的使用。
